# miR-34a-5p might have an important role for inducing apoptosis by down-regulation of SNAI1 in apigenin-treated lung cancer cells

**DOI:** 10.1007/s11033-021-06255-7

**Published:** 2021-03-06

**Authors:** Rieko Aida, Keitaro Hagiwara, Kazunori Okano, Kyoko Nakata, Yuho Obata, Takahiro Yamashita, Kaoru Yoshida, Hiromi Hagiwara

**Affiliations:** 1 Faculty of Biomedical Engineering, Toin University of Yokohama, 1614 Kurogane-cho, Aoba-ku, Yokohama, 225-8503 Japan; 2 Division of Materials Science, Nara Institute of Science and Technology, 8916-5 Takayama, Ikoma, 630-0192 Japan

**Keywords:** Apigenin, Human lung cancer cell A549, miR-34a-5p, SNAI1 (SNAIL), Apoptosis

## Abstract

Apigenin is a flavonoid with antioxidant and anticancer effects. It has been reported that apigenin inhibits proliferation, migration, and invasion and induces apoptosis in cultured lung cancer cells. However, there is little information on the involvement of microRNAs (miRNAs) in its effects. miRNA microarray analysis and polymerase-chain-reaction analysis of miRNAs revealed that treatment of human lung cancer A549 cells with apigenin up-regulated the level of miR-34a-5p. Furthermore, mRNA microarray analysis and the results of three microRNA target prediction tools showed that Snail Family Transcriptional Repressor 1 (SNAI1), which inhibits the induction of apoptosis, had its mRNA expression down-regulated in A549 cells treated with apigenin. Our findings suggest that apigenin might induce apoptosis by down-regulation of SNAI1 through up-regulation of miR-34a-5p in A549 cells.

## Introduction

Apigenin (4′,5,7-trihydroxyflavone) is a flavone classed as a flavonoid based on the structure flavan, and is found in many kinds of vegetables and fruits [1] including olives [2], parsley [3, 4], celery [3, 5], chamomile [6] and guava [1]. Apigenin has antioxidant effects that stabilize free radicals of the reactive oxygen species that can damage DNA or proteins [7]. It also has anti-cancer effects that include inhibiting cell growth, arresting the cell cycle, and inducing apoptosis in many cancers including leukemia [8, 9]. It has been reported that these effects are due to many signaling pathways [10, 11] in a number of cancer cell lines including human lung cancer A549 cells [12]. However, there is little information on the involvement of miRNAs in its effects. In this study, we investigated the involvement of miRNA on the anti-cancer effects of apigenin in human lung cancer A549 cells.

miRNAs bind with sequence complementarity to the 3′ untranslated regions (3′UTR) of one or more target mRNAs and act as endogenous regulators of their gene expression [13]. miRNAs are first transcribed by RNA polymerase II in the nucleus to primary miRNAs (pri-miRNAs). Pri-miRNAs are then processed by class 2 ribonuclease III enzyme (Drosha) to generate precursor miRNAs (pre-miRNAs). Then, pre-miRNAs are exported into the cytoplasm by the transporter exportin-5 (XPO5) [14]. In the cytoplasm, pre-miRNAs are processed by RNAse III (Dicer) [15], generating mature miRNAs, which are a double-stranded and approximately 22 nucleotides in length without the stem loop structures. One of the two complementary short RNA molecules is integrated into the RNA-induced silencing complex (RISC complex) that contains members of the Argonaute (Ago) family and regulates mRNA expression by binding to imperfect complementary sites, mainly within the 3′UTR [16]. The section containing nucleotides 2 through 8 of the miRNAs 5′ end is called the seed region and dominates the binding process. miRNAs have a variety of crucial regulatory functions that are associated with various human diseases, including cancer [17–19]. To find mRNA targets of miRNAs, prediction tools are used, and each tool has different methods and algorithms.

## Materials and methods

### Cell culture

A549 cells were obtained from RIKEN Cell Bank (Tsukuba, Japan). Cells were cultured in Dulbecco’s Modified Eagle’s Medium (low glucose)(DMEM, SIGMA-ALDRICH, St. Louis, MO, USA), supplemented with 10% fetal bovine serum (Moregate BioTech, Bulimba, Australia), 1% penicillin and streptomycin, and 2% GlutaMax™ (GIBCO, Dublin, Ireland), and incubated in a humidified atmosphere of 5% CO_2_ at 37 °C. For caspase-3/7 activity assays, cells were seeded at a density of 5 × 10^3^ cells/cm^2^ in 35-mm glass bottom dishes (IWAKI, Japan) and treated with various concentrations of apigenin. For extracting total RNA, cells were seeded at a density of 1 × 10^5^ cells/well in 6-well plates and were treated with 50 μM apigenin or dimethyl sulfoxide (DMSO, as control) for 48 h. We chose this concentration (50 μM) and exposure time (48 h) based on the 50% lethal dose.

### Cell viability assay

A549 cells were seeded at a density of 700 cells/well in 96-well plates and were subcultured for 1 day. After cells were treated with various concentrations (0, 20, 40, 60, 80, and 100 µM) of apigenin for 72 h, cell viability was measured by using the Cell Counting Kit-8 (Dojindo, Japan), according to the manufacturer’s protocol. Viable cells have NADH/NADPH and dehydrogenase to produce ATP by glycolysis, while dead cells do not. Thus, viable and dead cells are identified by assaying NAD/NADPH, which is performed by coupling with a colorimetric tetrazolium redox reaction mediated by the electron carrier 1-methoxy-5-methylphenaziniummethylsulfate.

### Caspase-3/7 activity assay

A549 cells were seeded at a density of 5 × 10^3^ cells/cm^2^ in 35-mm glass-bottom dishes and were treated with 100 μM apigenin after being subcultured for 1 day. The activity of caspase-3/7 in A549 cells treated with apigenin for 72 h was visualized using the CellEvent™ Caspase-3/7 Green Detection Reagent (Invitrogen, Japan) according to the manufacturer’s protocol. Staurosporine at 10 µM was used as a positive control.

### miRNA microarray assay

Total RNA was isolated from A549 cells treated with 50 μM apigenin for 48 h using the miRNeasy mini kit (Qiagen, Hilden, Germany) following the manufacturer’s protocol. For miRNA expression analysis, the Human miRNA Microarray V21.0 array (based on miRbase release 21.0, Agilent Technologies, Inc., Santa Clara, CA, USA) was used according to the manufacturer’s protocol. Total RNA was labelled and hybridized using the miRNA Complete Labeling and Hyb Kit (Agilent Technologies, Inc.). The miRNA microarray chips were scanned using an Agilent SureScan MicroArray Scanner (G2600D, Agilent Technologies, Inc.), and the signal values were analyzed using Feature Extraction software 12.0.3.1 (Agilent Technologies, Inc.).

### PCR of miRNA

The expressions of miR-34a-5p in A549 cells treated with 50 μM apigenin for 48 h were examined by real-time qRT-PCR. Total RNA was isolated from apigenin-treated A549 cells using the miRNeasy mini kit (Qiagen) following the manufacturer’s protocol and 1 µg of the total RNA was reverse-transcribed to complementary DNA (cDNA) using the TaqMan MicroRNA Reverse Transcription Kit (Applied Biosystems, CA, USA) according to the manufacturer’s protocol. qRT-PCR analysis was performed using the TaqMan Universal PCR Master Mix II (Applied Biosystems) by a LightCycler 480 System II (Roche, Basel, Switzerland). This PCR was performed using specific primers: TaqMan MicroRNA Assays INV, S hsa-miR-34a and RNU6B as an endogenous control.

### mRNA microarray

We used the same total RNA that was used in the miRNA microarray. Total RNA was labelled with the Low Input Quick Amp Labelling Kit (Agilent Technologies, Inc.) and hybridized using the Gene Expression Hybridization Kit (Agilent Technologies, Inc.). The Sureprint G3 Human Gene Expression V3 array (26,083 Entrez Genes, 30,606 lncRNAs, Agilent Technologies, Inc.) was used according to the manufacturer's protocol. The chips were scanned using a SureScan MicroArray Scanner (G2600I, Agilent Technologies, Inc.), and the signal values were analyzed using Feature Extraction software 12.0.3.1 (Agilent Technologies, Inc.).

### Real-time RT-qPCR of mRNA

mRNA expression level of SNAI1 and FOXG1 in A549 cells treated with 50 μM apigenin for 48 h were examined by real-time qRT-PCR. Total RNA (1 μg) was reverse-transcribed using a Roche Transcriptor First Strand cDNA Synthesis Kit (Roche, IN, USA) according to the manufacturer’s protocol. qRT-PCR analysis was performed using Roche LightCycler 480 SYBR Green I Master (Roche) by a LightCycler 480 System II (Roche). Specific primers for real-time qRT-PCR were designed using the website of primer3plus and are as follows: SNAI1: sense 5′-ACCCCACATCCTTCTCACTG-3′ and antisense 5′-TACAAAAACCCACGCAGACA-3′, FOXG1: sense 5′-GTCAATGACTTCGCAGAGCA-3′ and antisense 5′-GTCTGGTCCCAGGGATGTTA-3′ and β-actin: sense 5′-GGA CTT CGA GCA AGA GAT GG-3′ and antisense 5′-AGC ACT GTG TTG GCT TAC AG-3′ (Eurofins Genomics, Tokyo, Japan).

### Prediction of target mRNAs

We searched target mRNAs of miR-34a-5p using three prediction web tools: TargetScan (http://www.targetscan.org/vert_72/), DIANA TOOLS (http://diana.imis.athena-innovation.gr/DianaTools/index.php?r=microT_CDS/index), and miRDB (http://mirdb.org), and made three lists. Genes in two or three lists were chosen as target mRNAs of miR-34a-5p. Results of the three prediction tools were merged into a predicted target mRNAs list with the data extraction and reporting tool, AWK in UNIX. These lists were combined with the list of decreasing mRNAs in apigenin-treated A549 cells by microarray analysis into the final target mRNAs list.

### Statistical analysis

Numerical data were expressed as mean ± SD values of the results from three observations and the significance of differences was analyzed by using two-sided Student’s *t*-test. Statistical significance was set at P < 0.05. Experiments were repeated independently in triplicate and the results were qualitatively identical in every case.

## Results

### Effects of apigenin on viability and apoptosis of A549 cells

**Fig. 1:**
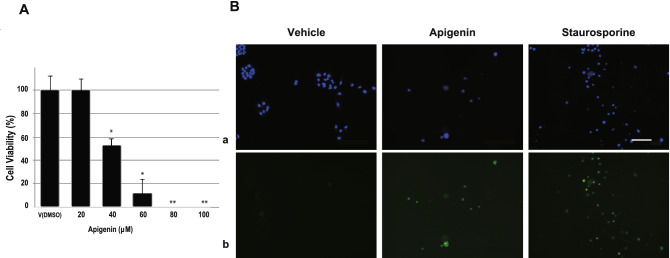
Effects of apigenin on viability and apoptosis of A549 cells. **A** A549 cells were treated by various concentrations of apigenin (n = 5) and cell viability was measured by using the Cell Counting Kit-8 (Dojindo, Japan). Data are representative of results from three separate experiments. *P < 0.05 *vs.* vehicle, and **P < 0.01 *vs.* vehicle. **B** Activity of caspase-3/7 in 100 μM apigenin-treated A549 cells for 72 h was visualized using the CellEvent™ Caspase-3/7 Green Detection Reagent (Invitrogen, Japan) with a fluorescence microscope (KEYENCE BZ-X800, Osaka, Japan). Photographs **a** and **b** show the cells stained with Hoechst 33,342 to visualize nuclei and with CellEvent Caspase-3/7 reagents to detect apoptosis, respectively. Staurosporine at 10 μM was added to dish for 3 h. Bar 100 μm

As shown in Fig. 1A, apigenin significantly decreased the viability of A549 cells in a dose-dependent manner. Forty micromolar apigenin was the 50% lethal dose for A549 cells and 80 μM was the lethal equivalent. Live cells were identified by nuclear staining (blue) with Hoechst 33,342 and caspase-3/7 activity (green) was fluorescently monitored as depicted in Fig. 1B a and b, respectively. Since apigenin strongly suppresses cell growth in a concentration as low as 50 μM, fewer cells were observed in the dish containing apigenin than in that of the vehicle control. Caspase-3/7 was activated in most cells remaining on the glass dish, equivalent to that of staurosporine-induced apoptosis.

### Up-regulation of miR-34a-5p by apigenin in A549 cells

**Fig. 2:**
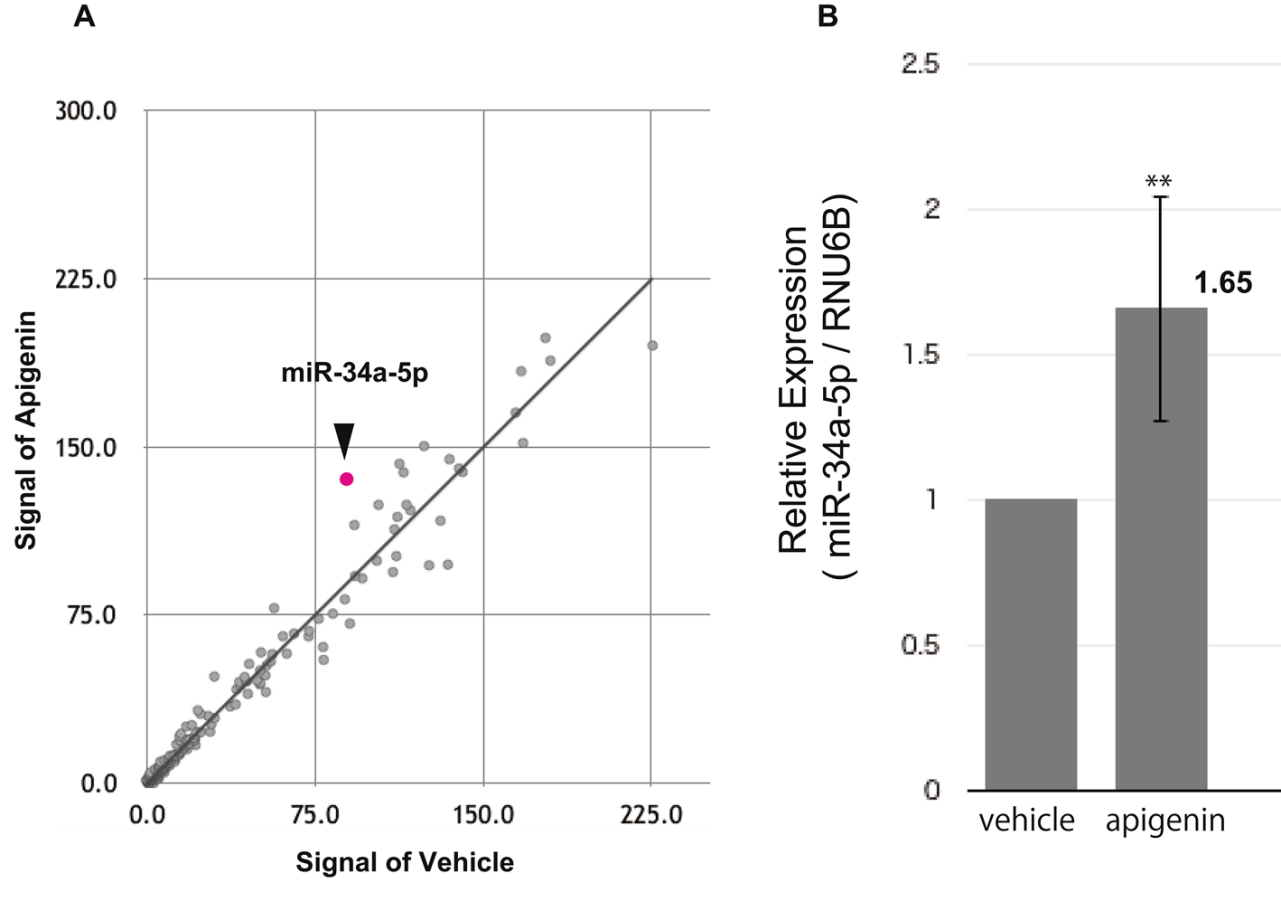
Up-regulation of miR-34a-5p by apigenin in A549 cells. **A** miRNA microarray analysis showed that miR-34a-5p was up-regulated by apigenin. A549 cells were treated with 50 µM apigenin for 48 h. **B** Up-regulation of miR-34a-5p was verified with real-time RT-qPCR (n = 3). **P < 0.01 *vs.* vehicle

To investigate the change in miRNA levels in apigenin-treated A549 cells, we performed miRNA microarray analysis (Fig. 2A). Consequently, miR-34a-5p was identified as a candidate that is up-regulated by 50 μM apigenin for 48 h in A549 cells. The expression of miR-34a-5p was increased by 1.53-fold against vehicle. To assess the expression level of miR-34a-5p quantitatively and to verify that up-regulation of miR-34a-5p was not a false-positive, we performed real-time RT-qPCR. As shown in Fig. 2B, expression of miR-34a-5p was significantly enhanced 1.65 ± 0.39-fold (n = 3) against vehicle, being in good agreement with the microarray analysis.

### Target mRNAs of miR-34a-5p

**Fig. 3:**
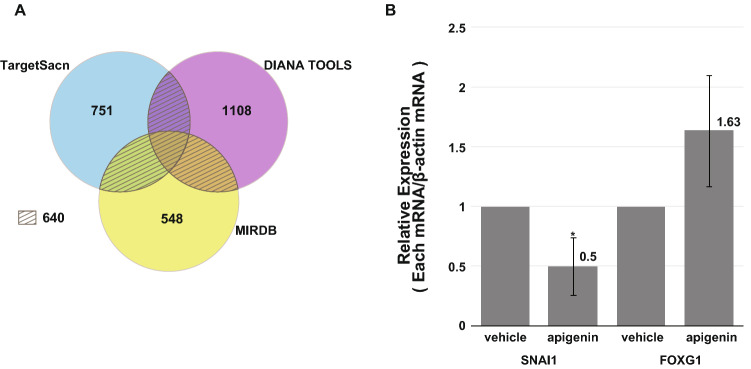
Down-regulation of SNAI1 mRNA by miR-34a-5p. **A** Venn diagram showing the overlap of mRNAs that were predicted to decrease by miR-34a-5p by alternative algorithms (TargetScan, DIANA TOOLS, and miRDB). **B** SNAI1 and FOXG1were chosen as targets of miR-34a-5p and were verified with real-time RT-qPCR (n = 3). *P < 0.05 *vs.* vehicle

To search for mRNAs decreased by miR-34a-5p in apigenin-treated A549 cells, we performed mRNA microarray analysis by using the same RNA sample that was used for the miRNA microarray. About 2000 mRNAs decreased to less than 0.5 times. To find mRNAs regulated by miR-34a-5p, we employed multiple algorithms, including TargetScan, DIANA TOOLS, and miRDB, to screen for specific mRNAs targeted by miR-34a-5p. TargetScan, DIANA TOOLS, and miRDB listed 751, 1108, and 547 targets, respectively. These analyses revealed 640 mRNAs included in two or more lists (Fig. 3A). From this list and the mRNA microarray, 32 target genes were selected (Table 1). Among the 32 mRNAs, we selected SNAI1 and FOXG1 mRNAs, because SNAI1 [20–22] and FOXG1 [23–25] have been reported to inhibit apoptosis. Real-time RT-qPCR revealed that the expression of SNAI1 mRNA in apigenin-treated A549 cells significantly decreased 0.50 ± 0.24-fold (n = 3) against the vehicle (Fig. 3B). FOXG1 was a false-positive (Fig. 3B).

**Table 1:** Candidates of target gene down-regulated by miR-34a-5p

Gene	Accession	Signal ratio
ANK3	NM 001204404	0.432
BMP3	NM 001201	0.475
C9orf47	NM 001001938	0.499
CA7	NM 001014435	0.386
CDH4	NM 991793	0.294
COL12A1	NM 004370	0.098
ESYT3	NM 031913	0.410
FAT4	NM 001291303	0.121
**FOXG1**	**NM 005249**	**0.444**
GABRA3	NM 000808	0.352
GPC6	NM 005708	0.491
HPSE	NM 006665	0.396
IGFBP3	NM 001013398	0.425
ISY1-RAB43	NM 001204890	0.465
MAPT	NM 016835	0.431
MARCH8	NM 001282866	0.251
MYOCD	NM 153604	0.099
NETO1	NM 138966	0.435
PARP15	NM 001113523	0.234
PDXK	ENST00000438837	0.352
RARB	NM 000965	0.279
RIMS3	NM 014747	0.447
SCN2B	NM 004588	0.136
SEMA4F	NM 004263	0.459
SERPINE1	NM 000602	0.499
SERPINF2	NM 000934	0.456
**SNAI1**	**NM 005985**	**0.447**
SNX30	NM 001012994	0.483
TANC2	NM 025185	0.491
TNFSF14	NM 003807	0.416
TRANK1	NM 014831	0.276
ZC3H12B	ENST00000617377	0.245

We selected bold genes as candidate

## Discussion

**Fig. 4:**
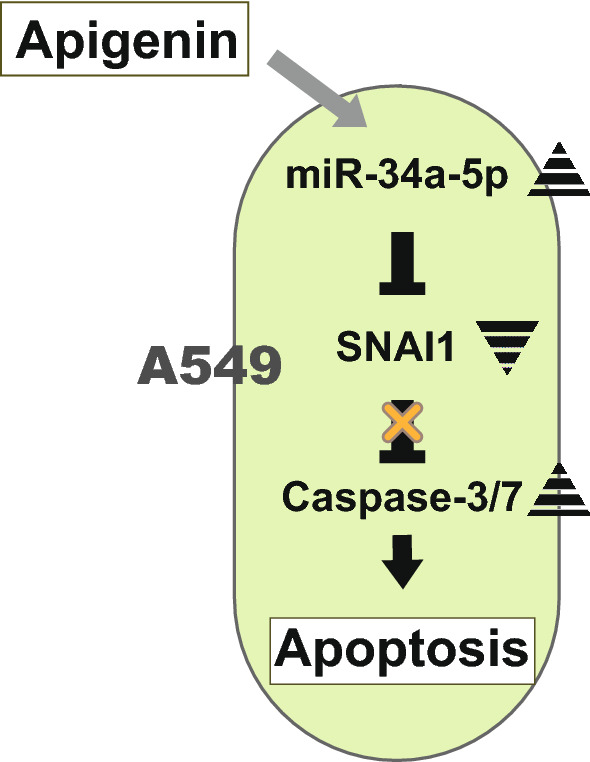
Schematic diagram by which the miR-34a-5p target SNAI1 regulates apoptosis with apigenin in A549 cells

In the present study, we tried to clarify the relationship between miRNA and apoptotic induction by apigenin in the lung cancer cell line A549. An miRNA micro array assay using A549 cells treated with apigenin revealed that expression level of miR-34a-5p increased in A549 cells. miR-34a-5p has been shown to target various genes involved in proliferation, metastasis and apoptosis [26–31]. The mRNA micro array assay and a merged list generated by three prediction tools suggested 32 miR-34a-5p candidate target genes. We selected SNAI1 and FOXG1 as candidates for inducers of apoptosis, because apigenin induced caspase-3/7 activity in A549 cells (Fig. 1B). Real-time RT-qPCR showed that level of SNAI1 mRNA decreased by 50% after apigenin treatment. Based on these results, we propose that apigenin induces apoptosis through the miR-34a-5p/SNAI1 pathway in A549 cells (Fig. 4). However, further investigation, for example, Western Blot analysis of SNAI1, is required in order to verify the SNAIL1 involvement.

Apigenin is well-known to have anti-cancer effects against a number of human cancer cells [9, 10], including human lung cancer A549 cells [32, 33], and is a potent remedial tool in cancer therapy [8]. Sung et al. [9] reported that the oral administration of apigenin (20–50 μg/mice) for 20 weeks reduces tumor volumes and induces complete abolishment of distant organ metastases in a transgenic adenocarcinoma of mouse prostate (TRAMP) model. These values are equivalent to 60–150 mg/60 kg when converted to human use. Apigenin is reported to exist at concentrations of 192–2408, 2000, 139, 3000–5000, and 579 mg/kg in olive leaf [2], parsley [9], celery leaf [5], chamomile [9], and guava [1], respectively. It may be possible to receive the required amount of apigenin from foods for the prevention of cancer.

Furthermore, apigenin is able to induce apoptosis in human lung cancer A549 cells in vitro [32, 33]. Recent reports show that miR-34a-5p is an inducer of apoptosis, cell-cycle arrest, and senescence in different cancers [27, 31]. Luteolin, which is also a flavone, induces apoptosis by up-regulation of miR-34a-5p in human gastric cancer cells [34]. SNAI1, a zinc-finger transcription factor, is known to mediate the enhancement of proliferation and the inhibition of apoptosis in cancer cells [35]. Moreover, Shenas et al*.* [36] demonstrated that the silencing of SNAI1 leads to the induction of apoptosis, and Wan et al*.* [22] revealed that inhibition of SNAIL enhances TRAIL-induced apoptosis. However, the stimulator of the miR-34a-5p /SNAI1 pathway in the apoptosis of A549 cells has not been clearly identified. In this study, we found that apigenin induces apoptosis through down-regulation of SNAI1 by up-regulating miR-34a-5p in lung cancer cells. Further research is needed to identify how apigenin up-regulates miR-34a-5p.
